# Supplementary material for: Integrative multi-omics analysis uncovers tumor-immune-gut axis influencing immunotherapy outcomes in ovarian cancer
Source: Nat Commun. 2024 Dec 5;15:10609. doi: 10.1038/s41467-024-54565-8 (PMC11621351; doi:10.1038/s41467-024-54565-8)
Supplement: Supplementary file 1 — Supplementary Information [file 41467_2024_54565_MOESM1_ESM.pdf]

Integrative Multi-Omics Analysis Uncovers Tumor-Immune-Gut Axis Influencing Immunotherapy Outcomes in Ovarian Cancer

Supplementary Figures:

Supplementary Figure 1. Updated clinical trial results and distribution of patients with Durable Clinical Benefit and Limited Clinical Benefit, based on clinical outcomes.

Supplementary Figure 2. Immune deconvolution analysis.

Supplementary Figure 3. Digital Spatial Profiling ROIs for patients.

Supplementary Figure 4. DSP principal component analysis.

Supplementary Figure 5. Microbiome diversity data.

Supplementary Figure 6. Stool microbiome bacterial differential OTU abundance associated with different time points and therapeutic response.

Supplementary Figure 7. Tumor microbiome bacterial differential OTU abundance limited, but associated with inflammatory and metabolic alteration.

Supplementary Figure 8. Serum metabolomics demonstrates differential circulating metabolites in LCB and DCB populations, from similar pathways as the fecal metabolites.

Supplementary Figure 9. Immune hot phenotype associated with specific metabolic and bacterial species.

Supplementary Figure 10. Immune, bacterial and metabolomics correlation network.

Supplementary Figure 11. Correlations of Dielma fastidiosa OTU with immune deconvolution scores demonstrate more significant relationship with T-cells, as compared to other immune cell populations.

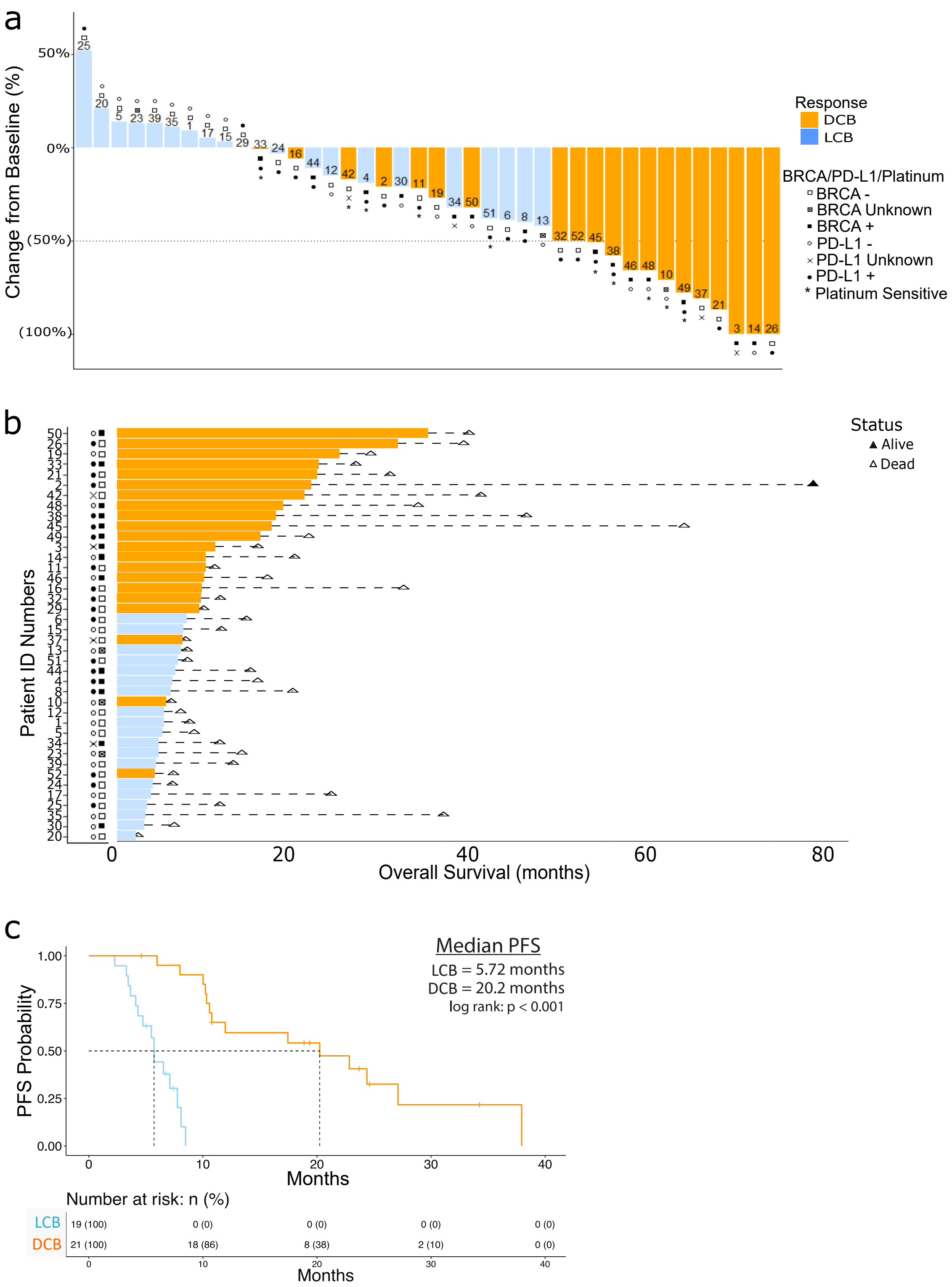

Supplementary Figure 1. Updated clinical trial results and distribution of patients with Durable Clinical Benefit and Limited Clinical Benefit, based on clinical outcomes. (a) Waterfall plot displays change in tumor size from baseline in durable clinical benefit (DCB, orange) and limited clinical benefit (LCB, blue) patient populations, with indications of BRCA, PD-L1, and Platinum Sensitivity Status. (b) Swimmer's plot details progression free survival (PFS) differences in these patient populations and their BRCA1 and PD-L1 status, indicating patients who are still alive (black triangle), and those that have died (white triangle). (c) Kaplan-Meier plot shows the difference in PFS between DCB (orange, median PFS = 20.2 months) and LCB (blue, median PFS = 5.72 months) populations.

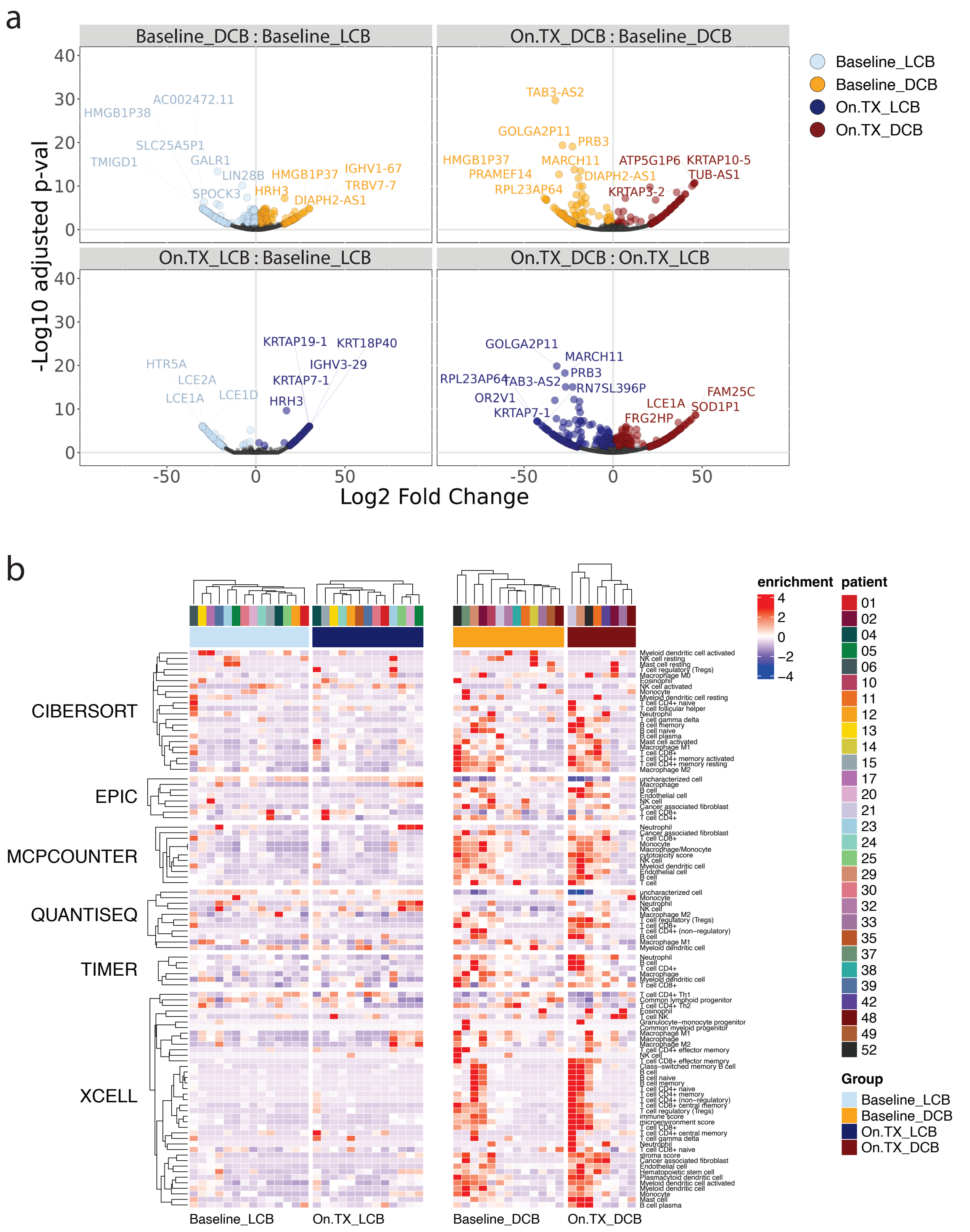

Supplementary Figure 2 Immune deconvolution analysis. (a) Volcano plots highlighting significant (|logFC|>1.5, adjusted $p < 0.05$; NS non-significant) differentially expressed genes (DEGs) between baseline LCB (Baseline_LCB, light blue, n=14), baseline DCB (Baseline_DCB, orange, n=13), on-treatment LCB (On-TX_LCB, dark blue, n=13), and on-treatment DCB (On-TX_DCB, red, n=8) patient populations. (b) Enrichment of 6 different immune deconvolution signatures in DCB patient populations compared to LCB patients at baseline and On-TX.

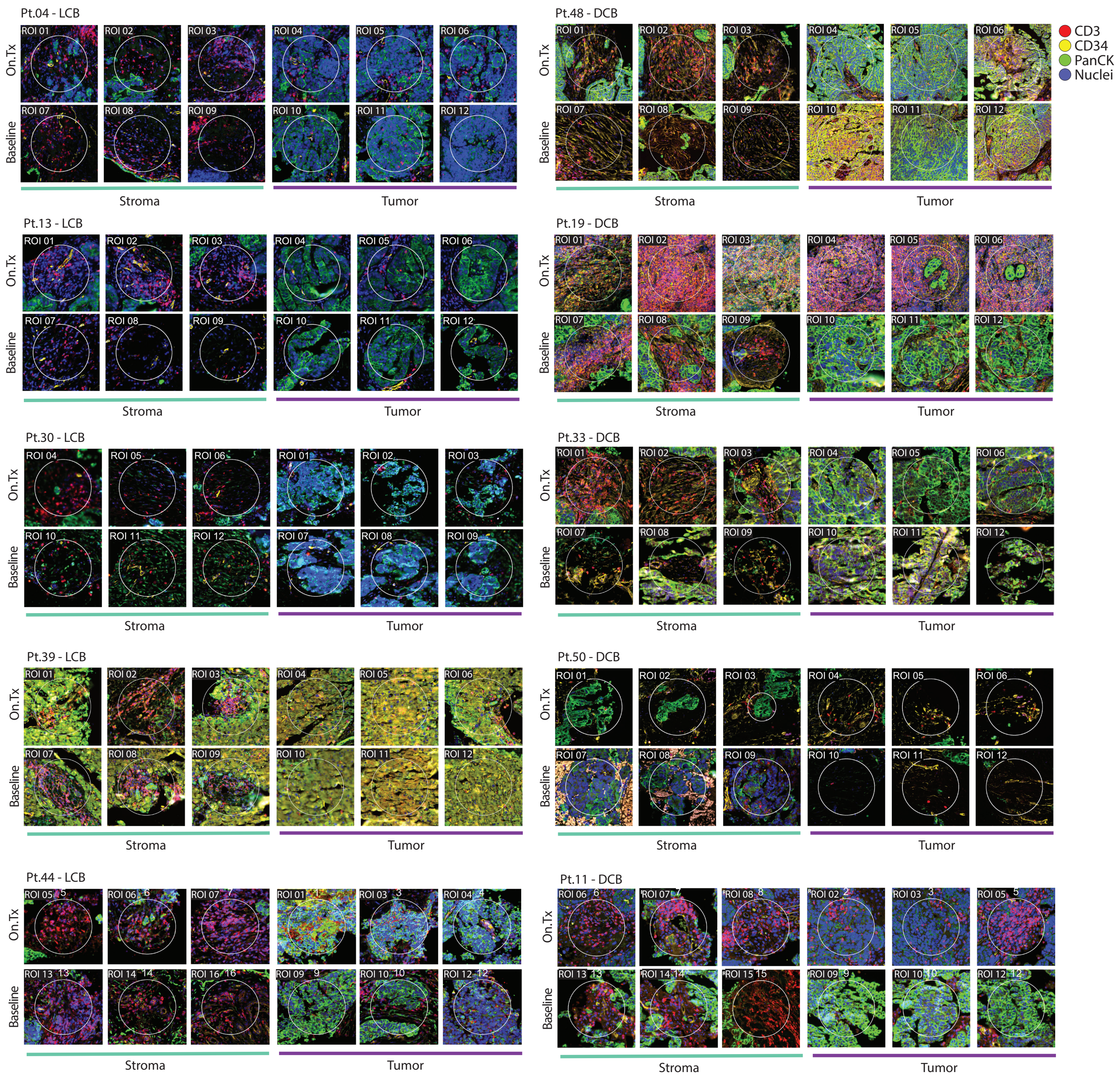

Supplementary Figure 3. Digital Spatial Profiling ROIs for patients. Representative images of ROIs from both durable clinical benefit and limited clinical benefit patients, used for DSP analysis. (blue = nuclei, green = PanCK, yellow = CD34, red = CD3)

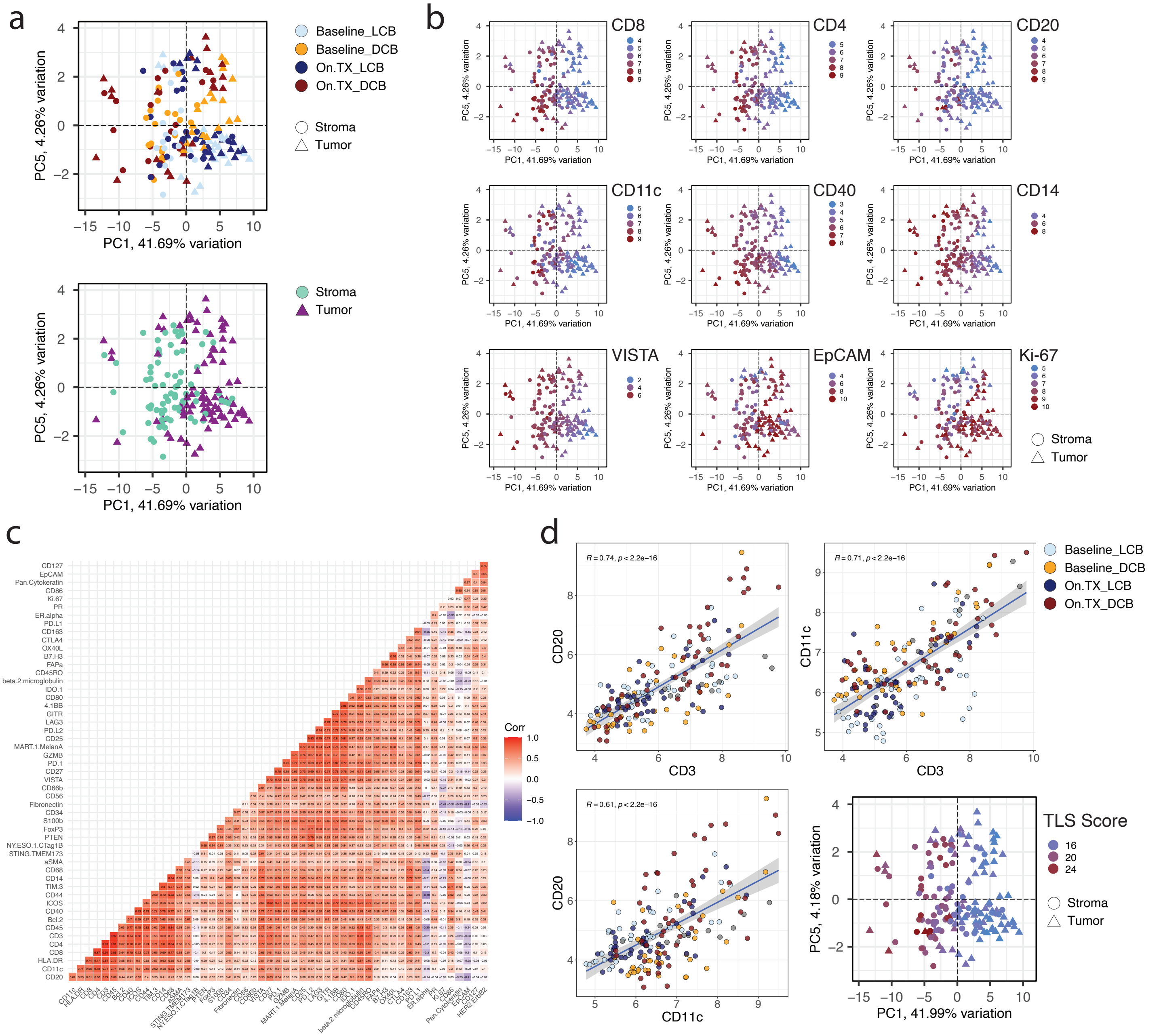

Supplementary Figure 4. DSP principal component analysis. (a) Principal component analysis (PCA) demonstrates separation of DCB and LCB patient populations based on all DSP markers (top), as well as stronger separation by location of samples in either the tumor (purple triangles) or stroma (teal circles), regardless of population (bottom). (b) Abundances of select immune (CD8, CD4, CD20, CD11C, CD40, CD14, VISTA), epithelial (EPCAM), and proliferative (Ki-67) markers mapped in PCA space, highlighting heterogeneity in ROI immune infiltration. (c) Correlation matrix of all detected markers across all 168 ROI, revealing highly coordinated features across tissue regions. (d) Correlations for T (CD3), B (CD20), and DC (CD11C) markers across ROI (Pearson r is shown). Summed abundance of these 3 lineage markers were used to estimate a TLS score (lower right). *p<0.05, **p<0.01, ***p<0.001; derived from linear regression analysis of abundances by clinical group, correcting for the patient in paired analyses when appropriate.

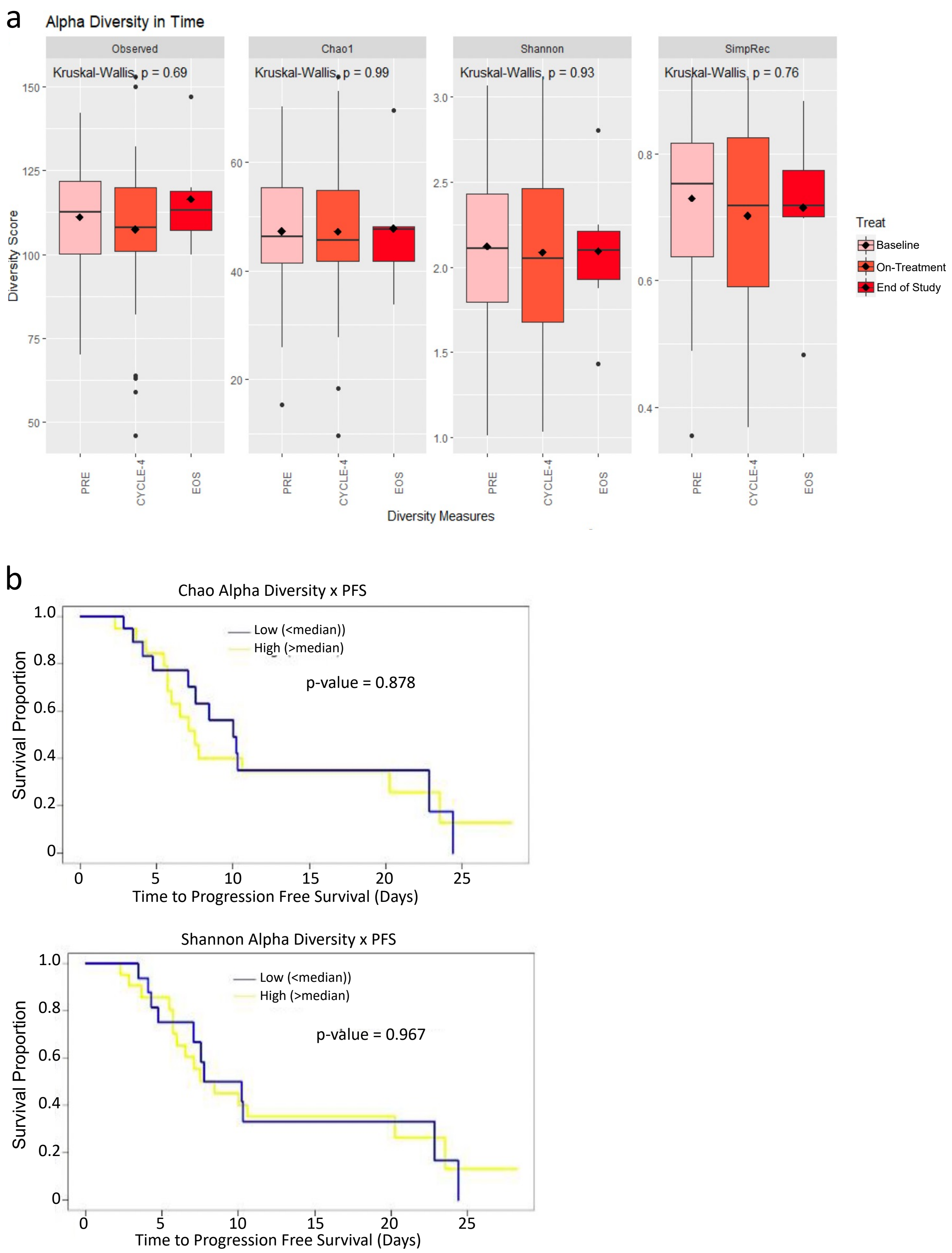

Supplementary Figure 5. Microbiome diversity data. (a) Alpha diversity and (b) alpha diversity by progression free survival (PFS) show no significant differences between durable clinical benefit (DCB) and limited clinical benefit (LCB) populations.

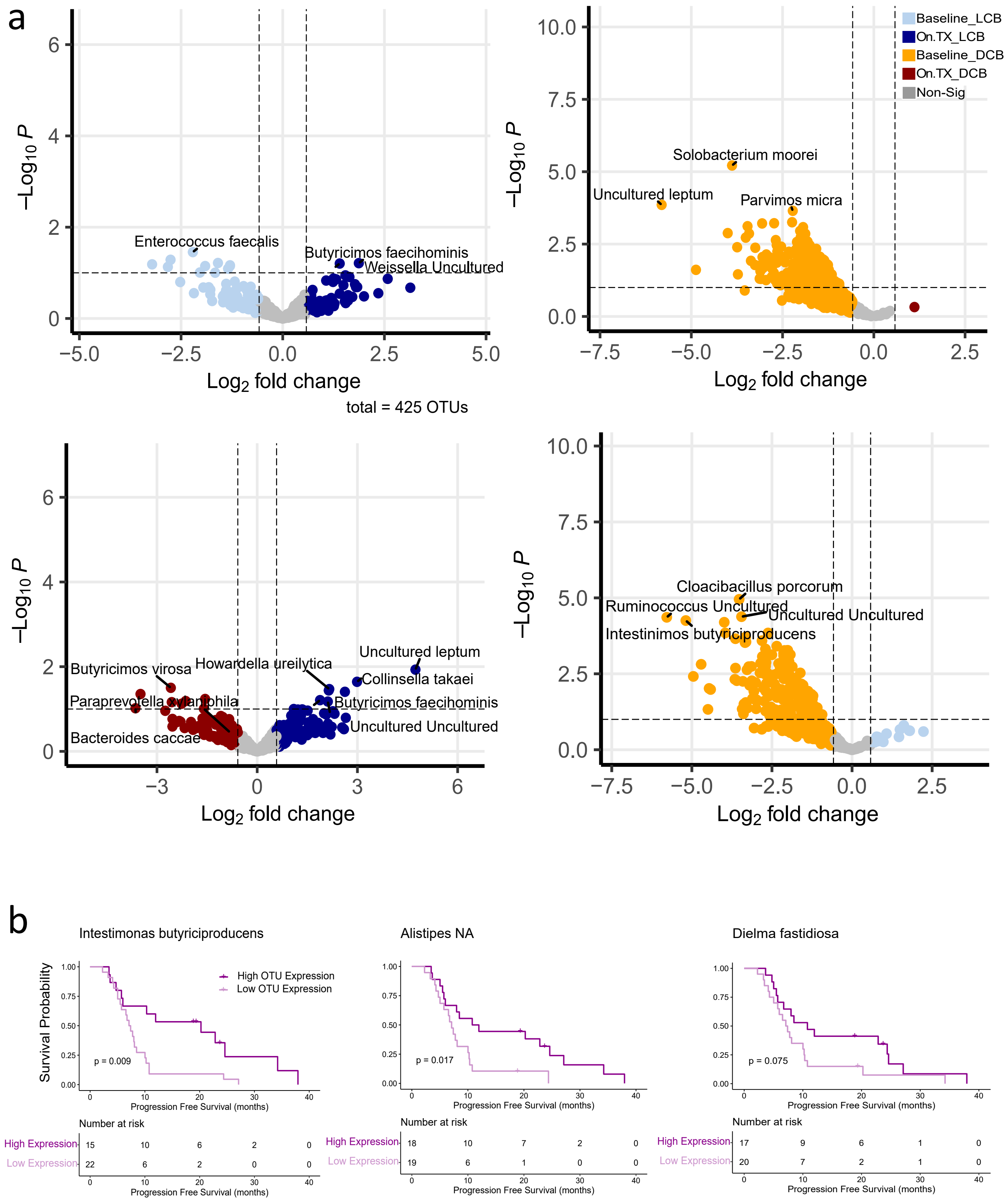

Supplementary Figure 6. Stool microbiome bacterial differential OTU abundance associated with different time points and therapeutic response. (a) Differential OTU abundance analysis comparing the four response groups resulted in unique microbes associated with each state, as demonstrated by the volcano plots. (b) Representative Kaplan-Meier plots demonstrate survival differences in patients associated with gut microbiota OTU abundance (split on median bacterial abundance, p-value indicative of log-rank p-value).

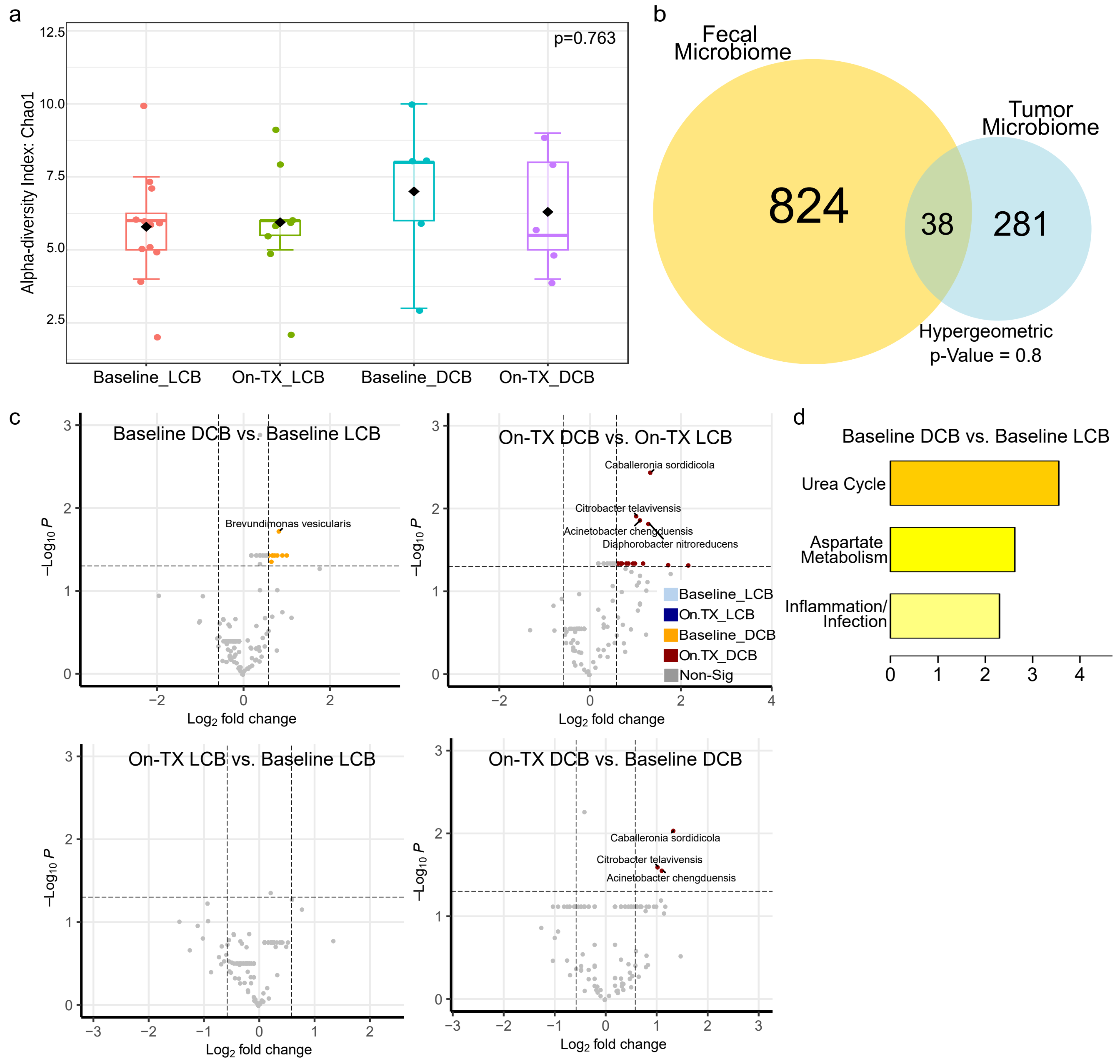

Supplementary Figure 7. Tumor microbiome bacterial differential OTU abundance limited, but associated with inflammatory and metabolic alteration. (a) Tumor microbiome, like the fecal microbiome, demonstrated no statistically significant differences in alpha diversity between Baseline LCB (n=12), On-TX LCB (n=12), Baseline DCB (n=5), and On-TX DCB (n=5) . (b) As expected, the tumor microbiome demonstrated limited biodiversity (n=319 species, blue), as compared to the fecal microbiome (n=862 species,yellow), with a limited and non-significant overlap between the two (n=38 species). (c) Differential OTU abundance analysis revealed no statistically significant changes by adjusted p value. Therefore, volcano plots demonstrate a pValue < 0.05 and |logFC| > 0.58. Overall, the comparison of Baseline DCB vs. Baseline LCB yielded 11 differentially abundant OTUs, On-TX DCB vs. On-Tx LCB yielded 18 differentially abundant OTUs, On-Tx DCB vs. Baseline DCB yielded 3 differentially abundant OTUs, and On-TX LCB vs. Baseline LCB revealed no differentially abundant OTUs. (d) The functional consequences of these altered species at baseline (Baseline DCB vs. LCB), enriched for Urea Cycle Metabolism, Amino Acid Metabolism, and Inflammatory/Infection-associated pathways.

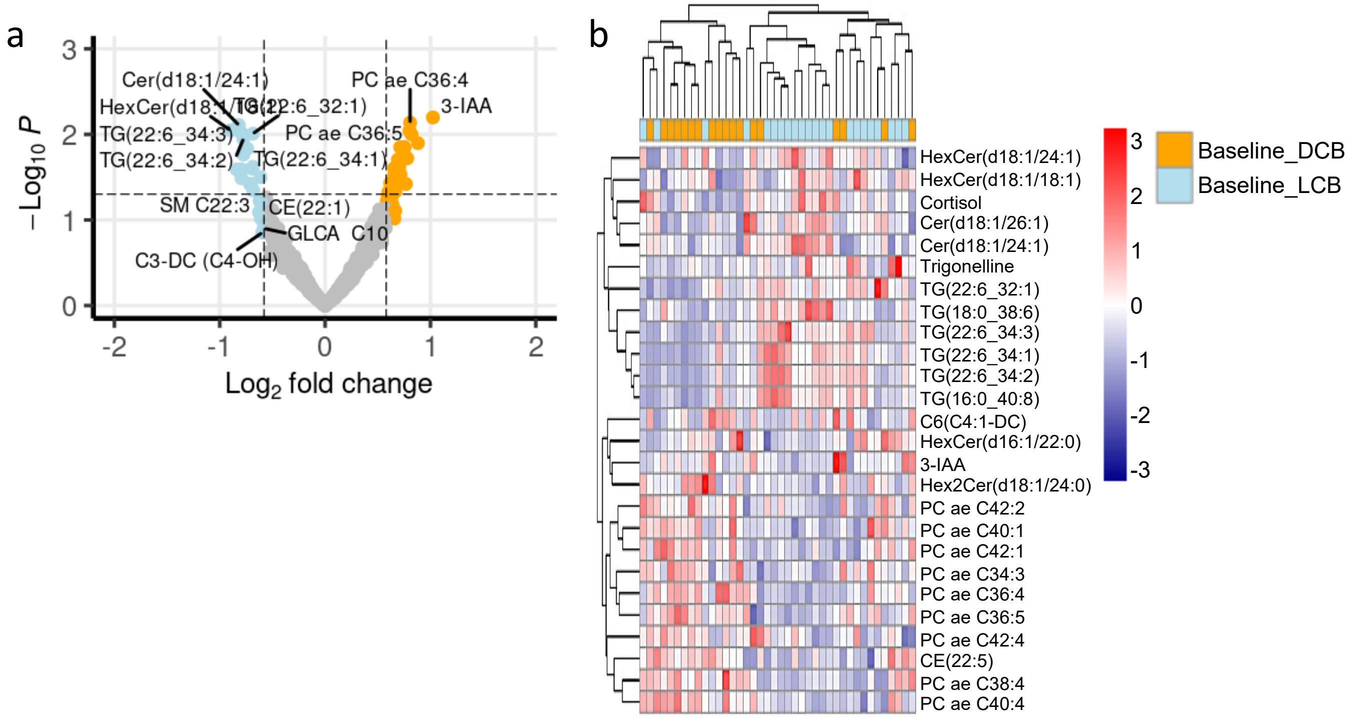

Supplementary Figure 8. Serum metabolomics demonstrates differential circulating metabolites in LCB and DCB populations, from similar pathways as the fecal metabolites. (a) Volcano plot demonstrates a large number of significantly differential metabolites between the LCB (n= 22, light blue) and DCB (n=18, orange) patients ($|logFC| > 1.5$, $p < 0.05$). The metabolite most highly upregulated in the DCB, being 3-IAA, a metabolite associated with tryptophan metabolism. (b) Heatmap similarly demonstrates increases in tryptophan metabolism (3-IAA) in DCB as compared to LCB, which may be indicative of increased catabolism. Conversely, phosphatidylcholines were decreased in the serum of DCB, as compared to LCB, which may indicate increased excretion of phosphatidylcholines, in patients with a durable clinical benefit.

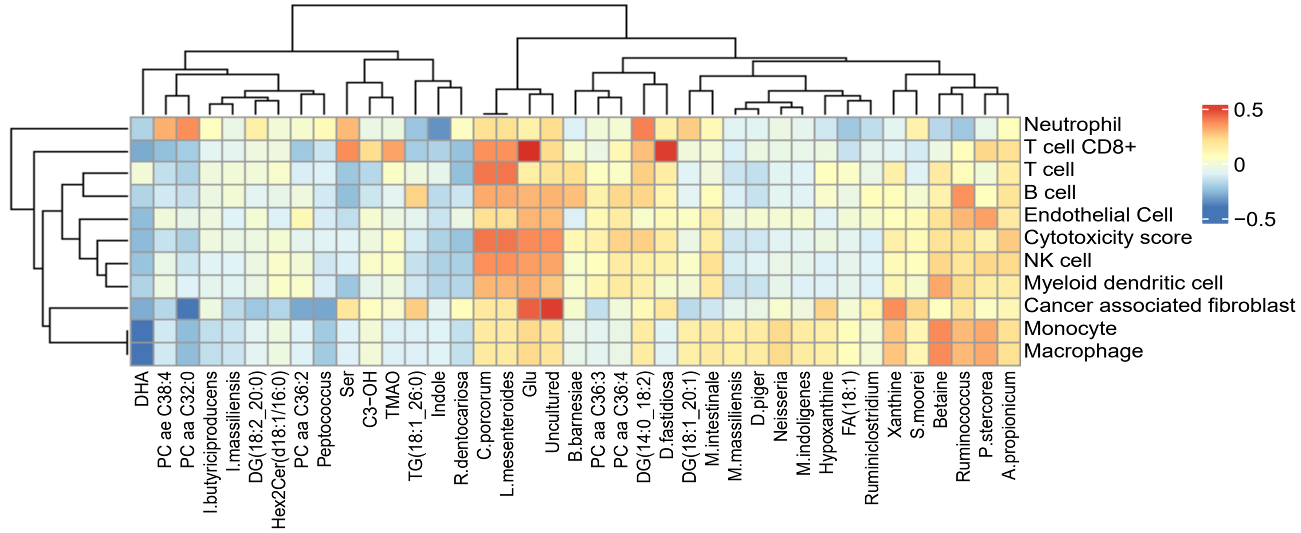

Supplementary Figure 9. Immune hot phenotype associated with specific metabolic and bacterial species. (a) Heatmap of Spearman correlation, across all patients, of patient fecal OTU abundance, as assigned by SILVA and NCBI, fecal metabolome, and tumor immune MCPCounter immune deconvolution category scores, resulted in distinct clusters of microbes and metabolites positively (red) or negatively (blue) correlated with immune signatures.

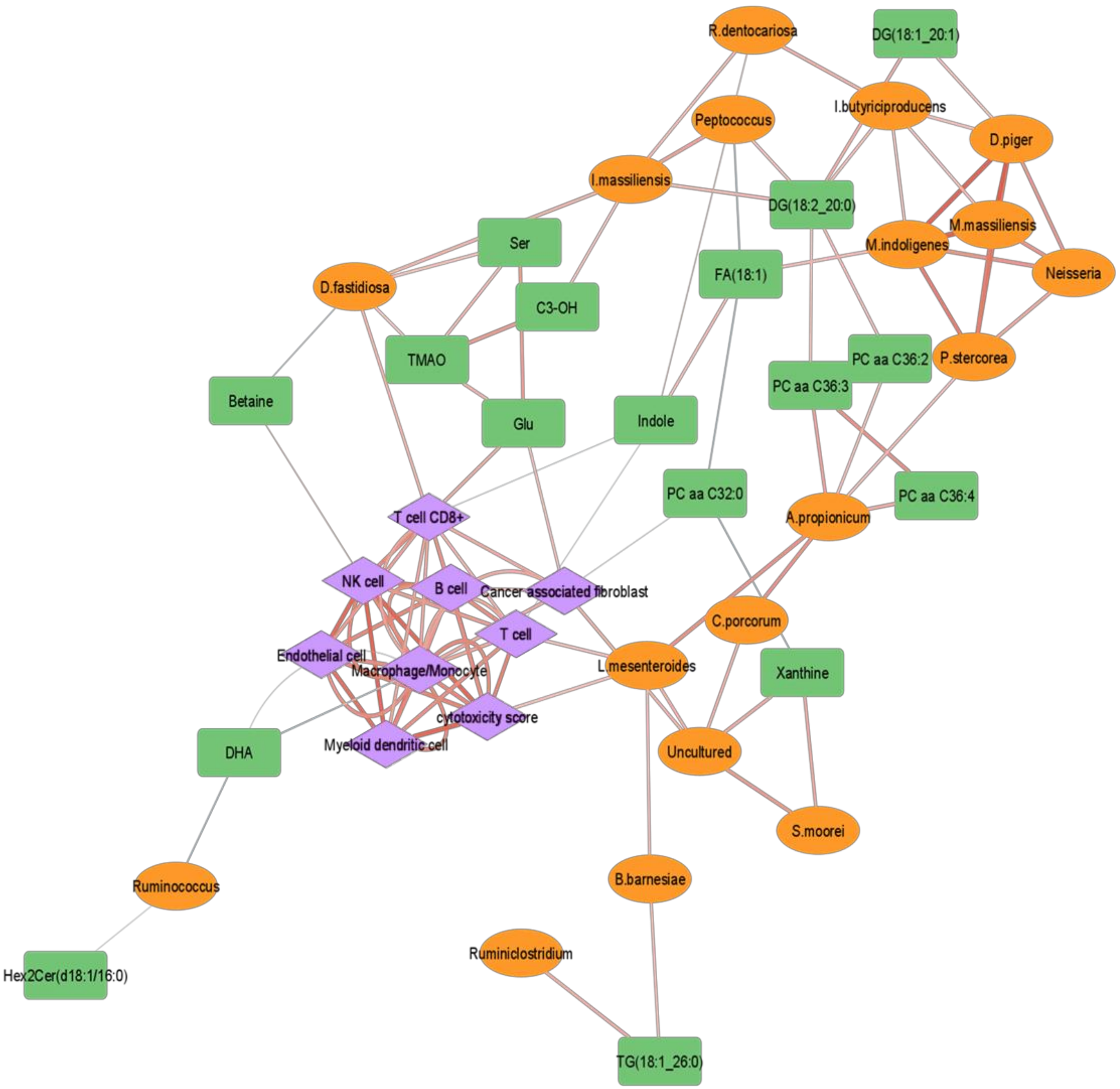

Supplementary Figure 10. Immune, bacterial and metabolomics correlation network. Correlation network of all significantly dysregulated metabolites (green rectangle), microbes (orange ellipses), and immune scores (purple diamond) nodes highly correlated with CD8+ T Cells, B cells, or Macrophages/Monocytes ($R^2 > 0.39$ (red), $R^2 < -0.39$ (blue), width of connectors increase with increased correlation value). Patient phenotype is designated by node borders with orange indicating the immune signature/microbe/metabolite was more highly expressed in DCB or blue for LCB.

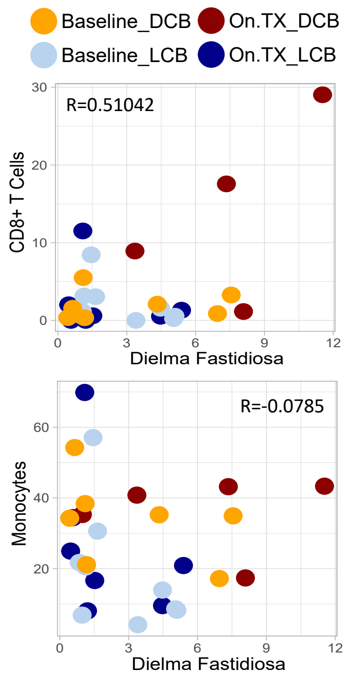

Supplementary Figure 11. Correlations of Dielma fastidiosa OTU with immune deconvolution scores demonstrate more significant relationship with T-cells, as compared to other immune cell populations. (a) Representative scatterplot of microbe (D. fastidiosa) with CD8+ T Cells (top), which are highly positively correlated, and monocytes (bottom, which are not). Points are shaded based on treatment timepoint and response.
