# Supplementary material for: Integrative multi-omics analysis uncovers tumor-immune-gut axis influencing immunotherapy outcomes in ovarian cancer
Source: Nat Commun. 2024 Dec 5;15:10609. doi: 10.1038/s41467-024-54565-8 (PMC11621351; doi:10.1038/s41467-024-54565-8)
Supplement: Supplementary file 3 — Reporting Summary [file 41467_2024_54565_MOESM3_ESM.pdf]

nature portfolio

Corresponding author(s): DBPR NCOMMS-24-32679-A

Last updated by author(s): October 14, 2024

Reporting Summary

Nature Portfolio wishes to improve the reproducibility of the work that we publish. This form provides structure for consistency and transparency in reporting. For further information on Nature Portfolio policies, see our Editorial Policies and the Editorial Policy Checklist.

Statistics

For all statistical analyses, confirm that the following items are present in the figure legend, table legend, main text, or Methods section.

n/a	Confirmed	
☐	☒	The exact sample size (*n*) for each experimental group/condition, given as a discrete number and unit of measurement
☐	☒	A statement on whether measurements were taken from distinct samples or whether the same sample was measured repeatedly
☐	☒	The statistical test(s) used AND whether they are one- or two-sided<br>*Only common tests should be described solely by name; describe more complex techniques in the Methods section.*
☐	☒	A description of all covariates tested
☐	☒	A description of any assumptions or corrections, such as tests of normality and adjustment for multiple comparisons
☐	☒	A full description of the statistical parameters including central tendency (e.g. means) or other basic estimates (e.g. regression coefficient) AND variation (e.g. standard deviation) or associated estimates of uncertainty (e.g. confidence intervals)
☐	☒	For null hypothesis testing, the test statistic (e.g. *F*, *t*, *r*) with confidence intervals, effect sizes, degrees of freedom and *P* value noted<br>*Give P values as exact values whenever suitable.*
☒	☐	For Bayesian analysis, information on the choice of priors and Markov chain Monte Carlo settings
☐	☒	For hierarchical and complex designs, identification of the appropriate level for tests and full reporting of outcomes
☐	☒	Estimates of effect sizes (e.g. Cohen's *d*, Pearson's *r*), indicating how they were calculated

Our web collection on statistics for biologists contains articles on many of the points above.

Software and code

Policy information about availability of computer code

Data collection	Data management activities for the clinical trial was performed using eClinical.
Data analysis	All clinical data analyses were conducted in SAS, version 9.4 (SAS Institute, Inc), and R version 3.6.1, which are purchased by Roswell Park.

For manuscripts utilizing custom algorithms or software that are central to the research but not yet described in published literature, software must be made available to editors and reviewers. We strongly encourage code deposition in a community repository (e.g. GitHub). See the Nature Portfolio guidelines for submitting code & software for further information.

Data

Policy information about availability of data

All manuscripts must include a data availability statement. This statement should provide the following information, where applicable:

- Accession codes, unique identifiers, or web links for publicly available datasets
- A description of any restrictions on data availability
- For clinical datasets or third party data, please ensure that the statement adheres to our policy

Raw and processed data derived from RNA-seq (GSE206422) are deposited at the NCBI Gene Expression Omnibus. Data from 16S rRNA-seq (PRJNA852556) are deposited at the NCBI Sequence Read Archive. Metabolomics data (Supplementary Table 2) are under review in the EMBL-EBI Metabolights database.

Human research participants

Policy information about studies involving human research participants and Sex and Gender in Research.

Reporting on sex and gender	This study reports on ovarian cancer, thus only women are included.
Population characteristics	Women with recurrent ovarian cancer were recruited to the clinical trial. For details on eligibility, please see the clinical trial protocol attached.
Recruitment	This is a single site phase 2 clinical trial and all patients were recruited from the Gynecologic Oncology Department at Roswell Park Comprehensive Cancer Center.
Ethics oversight	The Roswell Park Data and Safety Monitoring Board assessed the progress of the study, the safety data, and critical endpoints. This clinical trial has been reviewed and approved by the Roswell Park IRB in accordance with the requirements of 45 CFR 46, including its relevant Subparts, as well as ICH-GCP codes 3.1-3.4, which outline Responsibilities, Composition, Functions, and Operations, Procedures, and Records of the IRB.

Note that full information on the approval of the study protocol must also be provided in the manuscript.

Field-specific reporting

Please select the one below that is the best fit for your research. If you are not sure, read the appropriate sections before making your selection.

☒ Life sciences ☐ Behavioural & social sciences ☐ Ecological, evolutionary & environmental sciences

For a reference copy of the document with all sections, see nature.com/documents/nr-reporting-summary-flat.pdf

Life sciences study design

All studies must disclose on these points even when the disclosure is negative.

Sample size	Sample size calculation was done prior to the start of the clinical trial by Drs. Kristopher Attwood and Katy Wang, who are members of the Departments of Biostatistics and Bioinformatics. Details on the Statistical Methodology is available in the clinical trial protocol in Section 12.
Data exclusions	No data was excluded and data is presented on all the evaluable patients on clinical trial.
Replication	Follow up data on "real world experience" using this triple combination regimen on a different cohort of 55 patients with recurrent ovarian cancer was presented at ESMO 2022. That data confirmed similar efficacy of the triple combination. We have not repeated all the translational work on those cohort of women.
Randomization	Not applicable.
Blinding	Not applicable.

Reporting for specific materials, systems and methods

We require information from authors about some types of materials, experimental systems and methods used in many studies. Here, indicate whether each material, system or method listed is relevant to your study. If you are not sure if a list item applies to your research, read the appropriate section before selecting a response.

Materials & experimental systems

n/a	Involved in the study	
☒	☐	Antibodies
☒	☐	Eukaryotic cell lines
☒	☐	Palaeontology and archaeology
☒	☐	Animals and other organisms
☐	☒	Clinical data
☒	☐	Dual use research of concern

Methods

n/a	Involved in the study	
☒	☐	ChIP-seq
☒	☐	Flow cytometry
☒	☐	MRI-based neuroimaging

Clinical data

Policy information about clinical studies
All manuscripts should comply with the ICMJE guidelines for publication of clinical research and a completed CONSORT checklist must be included with all submissions.

Clinical trial registration	ClinicalTrials.gov Identifier: NCT02853318
Study protocol	Provided to the Editor
Data collection	Data collection is described in details in the clinical trial protocol in Section 14.
Outcomes	Please see Sections 3 and 4 in the clinical trial protocol that describes all the outcomes.
