# Supplementary material for: Integrative multi-omics analysis uncovers tumor-immune-gut axis influencing immunotherapy outcomes in ovarian cancer
Source: Nat Commun. 2024 Dec 5;15:10609. doi: 10.1038/s41467-024-54565-8 (PMC11621351; doi:10.1038/s41467-024-54565-8)
Supplement: Supplementary file 4 — Description of Additional Supplementary Files [file 41467_2024_54565_MOESM4_ESM.pdf]

Description of Additional Supplementary Files

Supplementary Data 1: RNA-Sequencing Differentially Expressed Gene Results

Supplementary Data 2: 16S RNA-Sequencing Differentially Expressed Gene Results

Supplementary Data 3: Metabolomics Differentially Expressed Gene Results
